# Miyako *Bidens pilosa* Extract Ameliorates Allodynia and Suppresses Spinal Microglial Activation in Mice with Partial Sciatic Nerve Ligation

**DOI:** 10.3390/cimb47060453

**Published:** 2025-06-12

**Authors:** Ai Takahashi, Hiroko Miyagishi, Komugi Tsuruta, Hiroshi Nango, Dai Hirose, Yuri Aono, Minoru Tanigawa, Katsushi Nishimura, Minoru Saito, Takayuki Kawato, Tadashi Saigusa, Yasuhiro Kosuge

**Affiliations:** 1 Laboratory of Pharmacology, School of Pharmacy, Nihon University, 7-7-1 Narashinodai, Funabashi-shi 274-8555, Chiba, Japan; phai22002@g.nihon-u.ac.jp (A.T.); miyagishi.hiroko@nihon-u.ac.jp (H.M.); phko22004@g.nihon-u.ac.jp (K.T.); nango.hiroshi@nihon-u.ac.jp (H.N.); 2 Laboratory of Medical Microbiology, School of Pharmacy, Nihon University, 7-7-1 Narashinodai, Funabashi-shi 274-8555, Chiba, Japan; hirose.dai@nihon-u.ac.jp; 3 Department of Pharmacology, School of Dentistry at Matsudo, Nihon University, 2-870-1 Sakaechonishi, Matsudo-shi 271-8587, Chiba, Japan; aono.yuri1@nihon-u.ac.jp (Y.A.); saigusa.tadashi@nihon-u.ac.jp (T.S.); 4 Department of Materials and Applied Chemistry, College of Science and Technology, Nihon University, Building No. 2, 1-5-1 Kanda Surugadai, Chiyoda, Tokyo 101-0062, Japan; tanigawa.minoru@nihon-u.ac.jp; 5 Department of Science and Manufacturing Technology, Junior College, Nihon University, 7-24-1 Narashinodai, Funabashi-shi 274-8501, Chiba, Japan; nishimura.katsushi@nihon-u.ac.jp; 6 Department of Biosciences, College of Humanities and Sciences, Nihon University, 3-25-40 Sakurajosui, Setagaya-ku, Tokyo 156-8550, Japan; saitou.minoru79@nihon-u.ac.jp; 7 Department of Oral Health Sciences, Nihon University School of Dentistry, 1-8-13 Kanda-Surugadai, Chiyoda-ku, Tokyo 101-8310, Japan; kawato.takayuki@nihon-u.ac.jp

**Keywords:** neuropathic pain, Miyako *Bidens pilosa*, microglia, lumbar spinal cord

## Abstract

Neuropathic pain, characterized by chronic allodynia, remains difficult to manage with current pharmacotherapies. Microglial activation plays a pivotal role in the development and maintenance of neuropathic pain and represents a promising therapeutic target. We previously demonstrated that Miyako *Bidens pilosa* extract powder (MBP), derived from Miyako Island, Okinawa, suppresses glial activation in a mouse model of amyotrophic lateral sclerosis. In this study, we investigated the analgesic potential of MBP in a mouse model of neuropathic pain. Neuropathic pain was induced in male ICR mice by partial sciatic nerve ligation (PSNL). Mice were orally administered MBP (2 g/kg) or vehicle daily. Mechanical allodynia was assessed using von Frey filaments. On postoperative day 7, MBP-treated mice exhibited significantly reduced allodynia compared to vehicle-treated mice. MBP also attenuated thermal hyperalgesia on postoperative day 7. Lumbar spinal cords (L5) were subjected to immunohistochemical analysis for ionized calcium-binding adaptor molecule 1 (Iba1), a microglial marker. MBP significantly decreased the number of Iba1-positive microglia in the ipsilateral dorsal horn. These results suggest that MBP alleviates neuropathic pain, at least in part, by suppressing microglial activation in the spinal cord. MBP may represent a novel plant-derived therapeutic candidate for treating neuropathic pain.

## 1. Introduction

Nociception is an important sensory process triggered by harmful stimuli in the nervous system. Signals originating from peripheral tissues are transmitted to the brain via primary afferent fibers and spinal dorsal horn (SDH) neurons, which are often interpreted as pain signals. Acute nociceptive pain is a crucial protective mechanism that helps organisms avoid injury or harmful stimuli. In contrast, chronic pain can persist long after the initial injury has healed or even in the absence of any ongoing threat. Neuropathic pain is a severe and persistent form of chronic pain that can be caused by conditions such as diabetic neuropathy, postherpetic neuralgia, spinal cord injury, stroke, and various other neurological disorders [1]. Despite advancements in pain management, neuropathic pain remains difficult to treat because it is often resistant to standard analgesics. Current treatments aim to manage symptoms through various pharmacological and non-pharmacological interventions [2]. Moreover, many patients with neuropathic pain do not achieve sufficient relief from existing medications or experience side effects, which limits the effectiveness of current therapeutic strategies. Therefore, further research is required to identify new therapeutic targets based on clear pathological mechanisms.

Mechanical allodynia, a key feature of neuropathic pain, significantly reduces the quality of life by causing harmless tactile stimuli to be perceived as pain [3]. This phenomenon is associated with dysregulated signaling within the SDH network [4], as indicated by increased neuronal responses to light touch in mice with spared nerve injury-induced neuropathic pain [5]. Recent findings indicate that glial cells, including microglia and astrocytes, play key roles in the development and maintenance of neuropathic pain. Following peripheral nerve damage, these cells become activated and undergo morphological changes, proliferation, and expression of specific markers [6]. In particular, microglia play a crucial role in the development and maintenance of neuropathic pain and are potential therapeutic targets. Minocycline, an inhibitor of microglial activation, attenuates mechanical allodynia and hyperalgesia in a spinal nerve transection rat model of neuropathic pain [7]. Recently, pexidartinib (PLX-3397), a colony-stimulating factor-1 receptor (CSF-1R) inhibitor with microglia-depleting properties, was reported to ameliorate pain-related behavior in a CCI mouse model [8]. These findings highlight the involvement of microglia in neuropathic pain mechanisms and open new avenues for potential therapeutic targets, as current treatments often have limited efficacy and significant side effects. Despite these findings, there remains a need for alternative naturally derived treatments that can effectively modulate microglial activation with fewer side effects.

*Bidens pilosa* L. var. *radiata* SCHERFF (BP) is an annual herb of the Asteraceae family, originally native to South America, and is now widely distributed across tropical and subtropical regions worldwide [9]. In particular, enzymatic digestion of BP cultivated in the Miyako Islands of Okinawa Prefecture, Japan, known as Miyako BP extract (MBP), has been reported to exhibit anti-inflammatory [10] and antioxidant effects [11]. Moreover, MBP suppresses cell growth in human T-cell leukemia virus type 1-infected T-cell lines [12] and inhibits virus yield in RAW 264.7 cells infected with herpes simplex virus [13]. Our previous study demonstrated that oral administration of MBP prolongs the lifespan and protects motor neurons by inhibiting microglial activation in a mouse model of amyotrophic lateral sclerosis (ALS), a neurodegenerative disease that affects motor neurons [14]. In particular, we observed that MBP selectively suppressed the production of pro-inflammatory cytokines by controlling the polarization of M1 microglia in the lumbar spinal cord of an ALS mouse model [15]. Although microglial activation is involved in the pathogenesis of neuropathic pain, the effects of MBP have not yet been investigated. Therefore, this study aimed to evaluate the therapeutic potential of orally administered MBP in microglial activation and neuropathic pain induced by partial sciatic nerve ligation (PSNL) in mice. We hypothesized that MBP would reduce pain-related behaviors by attenuating microglial activation, thereby offering a new approach to neuropathic pain management.

## 2. Materials and Methods

### 2.1. Animals

Male ICR mice (Japan SLC Inc., Hamamatsu, Japan), weighing between 30 and 40 g, were kept in an environment maintained at 23 ± 1 °C with a 12 h light–dark cycle (lights on from 8:00 a.m. to 8:00 p.m.). They had unrestricted access to food and water. Before the experiments commenced, the mice underwent a one-week acclimatization period. The animals were randomly divided into the following groups: Sham, PSNL-water, and PSNL-MBP. A total of 62 mice, distributed across different experimental procedures, were used. Mice, in each group, were further allocated for behavioral analysis (Sham, *n* = 16; PSNL-water, *n* = 17; PSNL-MBP, *n* = 17) and immunohistochemistry experiments (Sham, *n* = 4; PSNL-water, *n* = 4; PSNL-MBP, *n* = 4). Although the researchers were aware of these group assignments, all assessments and analyses followed standardized procedures to minimize experimental bias. All animal procedures were performed in compliance with the ethical guidelines of the Nihon University Animal Care and Use Committee (Tokyo, Japan; experiment number: #AP22PHA011-1). Efforts were taken to alleviate animal suffering, reduce the total number of animals involved, and incorporate alternative methods into in vivo experimentation whenever possible.

### 2.2. Neuropathic Pain Model and MBP Treatment

The procedure used for the PSNL mice was based on a previously established protocol [16]. In summary, the right sciatic nerve was surgically exposed after the induction of isoflurane anesthesia. Approximately one-third to one-half of the nerve was ligated using an 8-0 catgut suture. In the sham-operated group, an identical surgical approach was used, but the nerve remained intact without ligation. All mice were weighed before (−1) and at 1, 3, 5, 7, 10, 12, 14, 17, 19, and 21 days after surgery.

**Figure 1:**
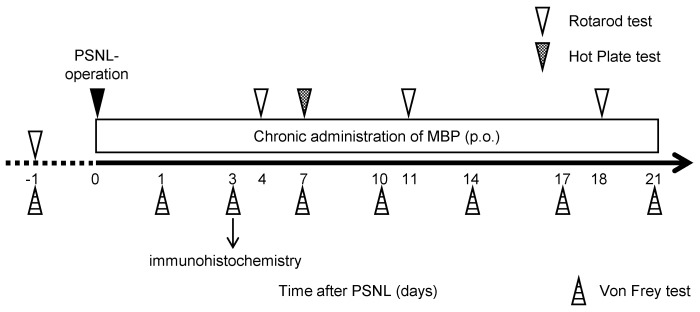
Schematic of the research protocol followed in this study.

MBP (brand name Musashino Miyako BP) was obtained from the Musashino Research Institute for Immunity (Miyako Island, Okinawa, Japan) and dissolved in injection-grade water (Otsuka, Tokyo, Japan) using a fresh solution prepared daily, as described previously [14]. Our research group has recently reconfirmed that MBP contains chlorogenic acid, caffeic acid, and rutin through high-performance liquid chromatography (HPLC) analysis [17]. In the present study, we employed the same lot of MBP that was used in the report by Fukuzawa et al. [17]. Immediately after recovery from postoperative anesthesia (day 0), MBP (2 g/kg/day) or vehicle (injection-grade water) was administered orally once daily using a disposable oral gavage syringe (Fuchigami, Kurume, Japan) until 21 days after surgery (Figure 1).

### 2.3. Mouse Motor Performance

Mouse motor function was assessed using a rotarod apparatus (Muromachi Kikai, Tokyo, Japan), according to a previously established method [14]. Mice were trained at a constant speed of 8 revolutions per minute (rpm), with a maximum duration of 300 s per trial, and the training was repeated until each mouse could remain on the rotarod for the full 300 s without falling. After the training period, mice remained in the apparatus. The maximum allowable rotarod duration at a speed of 8 revolutions per minute (rpm) was set at 300 s and recorded before (−1) and 4, 11, and 18 days after surgery. Observers who were blinded to MBP treatment performed the assessments simultaneously.

### 2.4. Assessment of Mechanical Withdrawal Thresholds

The mechanical withdrawal threshold (g) of the hind paw was assessed using von Frey filaments [16]. Briefly, each filament was applied perpendicular to the mid-plantar region of the hind paw with sufficient pressure to cause slight bending. The minimum force required to elicit a response such as paw lifting or licking was recorded as the withdrawal threshold. Withdrawal thresholds were measured at pre (−1), 1, 3, 7, 14, 17, and 21 days after surgery (Figure 1). Data were calculated as the average of the thresholds for both hind limbs tested three times at 10 s intervals.

### 2.5. Assessment of Thermal Hyperalgesia

A hot plate test was performed to measure the heat sensitivity. In brief, each mouse was placed on a hot plate at 55 °C and the reaction time for the appearance of signs of pain avoidance, such as jumping or paw licking, was noted. Animals that did not respond to the heat stimulus after 30 s were removed from the plate. The withdrawal latency of the right hind paw was measured three times at 5-min intervals, and the mean value was calculated and analyzed.

### 2.6. Immunohistochemistry

Immunohistochemistry (IHC) was performed as described previously [16,18]. Briefly, spinal cord tissue (L5) from 4 mice per treatment group was cut into 30 µm thick sections and incubated with rabbit anti-Iba-1 antibody (1:500; FUJIFILM Wako, #019-19741, Osaka, Japan) in Can Get Signal™ Immunostain Immunoreaction Enhancer Solution A (Toyobo, Osaka, Japan) at 4 °C for 72 h. After phosphate-buffered saline (PBS) washes, sections were treated with Alexa Fluor 488-conjugated goat anti-rabbit IgG secondary antibody (Molecular Probes, Eugene, OR, USA) at 1:250 dilution in the dark at room temperature for 2 h. Sections were mounted using VECTASHIELD^®^ HardSet mounting medium (Vector Laboratories, Newark, CA, USA). Quantification of Iba-1 expression was based on green immunoreactive area, including both microglial cell bodies and their processes. Immunofluorescence images of Iba-1-positive microglia were acquired using a fluorescence microscope (BZ-X800, Keyence, Osaka, Japan). The area of the immunostained region was automatically calculated using the Hybrid Cell Count application (BZ-H4C, Keyence, Osaka, Japan) in the BZ-X Analyzer software version 1.1.2 (BZ-H4A, Keyence, Osaka, Japan), with a consistent threshold applied across all samples.

### 2.7. Statistical Analysis

All statistical analyses were performed using GraphPad Prism version 9.5.1 software (GraphPad Software, La Jolla, CA, USA). Prior to analysis, data were tested for normality using the Shapiro–Wilk test. For datasets that followed a normal distribution, significant differences were analyzed using one-way or two-way analysis of variance (ANOVA), followed by Tukey’s test for multiple comparisons. For datasets that did not meet the assumptions of normality (e.g., immunohistochemistry results), non-parametric analysis was performed using the two-stage step-up method of Benjamini, Krieger, and Yekutieli. Results were expressed as the mean ± standard error (SE) or standard deviation (SD).

## 3. Results

### 3.1. MBP Had No Effect on Body Weight Gain and Motor Performance in PSNL Mice

**Figure 2:**
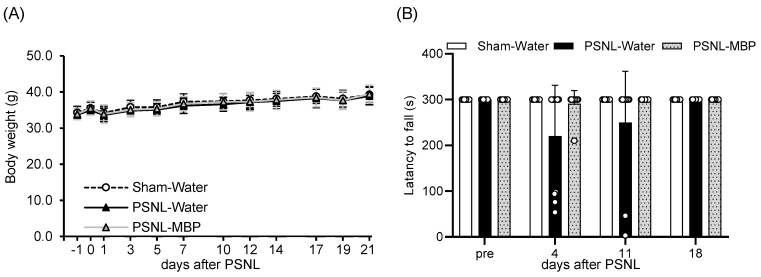
Time-course analysis of body weight changes and motor performance in Sham-Water, PSNL-Water, and PSNL-MBP groups over 21 days. (**A**) Body weight changes in the Sham-Water, PSNL-MBP, and PSNL-Water groups. Data are shown as mean ± SD, *n* = 12–13. Statistical comparisons were conducted using two-way ANOVA followed by Tukey’s test. (**B**) Motor performance of the mice was evaluated using a rotarod apparatus at 18 days after PSNL or sham surgery. Graph depicts latency to fall from the rotarod apparatus in Sham-Water, PSNL-MBP, and PSNL-Water groups. White circles represent individual values. Data are presented as mean ± SE, *n* = 12–13. Statistical analysis was conducted using one-way ANOVA followed by Tukey’s test.

The PSNL model has been widely used in neuropathic pain research. As pain progressed, a decline in appetite, reduced activity levels in the mouse models, and subsequent weight loss were observed at 10 weeks after PSNL surgery [19]. First, we examined the effects of MBP administration on body weight gain in PSNL mice. Mild weight gain was observed in all three groups (Sham-Water, PSNL-Water, PSNL-MBP), and no significant differences in weight gain were observed (day 21 PSNL-Water, *p* = 0.3536 vs. Sham, day 21 PSNL-MBP, *p* = 0.3264 vs. PSNL-water) (Figure 2A).

Contrastingly, rotarod analysis demonstrated that motor performance tended to decrease in the PSNL-Water mice compared to controls (Sham-Water mice) at day 4 and day 11 (day 4 PSNL-Water, *p* = 0.09 vs. Sham, day 4 PSNL-MBP, *p* = 0.1474 vs. PSNL-Water, day 11 PSNL-Water, *p* = 0.3381 vs. Sham, day 11 PSNL-MBP, *p* = 0.3381 vs. PSNL-Water). These trends of decreased motor performance in PSNL mice were attenuated by oral administration of MBP, but these changes were not significant (Figure 2B).

### 3.2. MBP Ameliorated the PSNL-Induced Mechanical Allodynia in Mice

**Figure 3:**
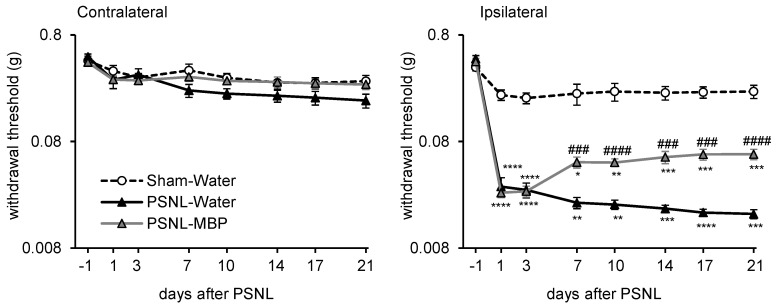
Time-course analysis of mechanical allodynia assessed by the von Frey test in PSNL mice. Contralateral side (left panel) and Ipsilateral side (right panel) mechanical withdrawal thresholds in Sham-Water, PSNL-MBP, and PSNL-Water groups over 21 days. Data are presented as mean ± SE, *n* = 12–13. Statistical analysis was conducted using two-way ANOVA followed by Tukey’s test. * *p* < 0.05, ** *p* < 0.01, *** *p* < 0.001, **** *p* < 0.0001 vs. Sham; ^###^ *p* < 0.001, ^####^ *p* < 0.0001 vs. PSNL-Water.

Next, we evaluated the analgesic effects of orally administered MBP on PSNL mice. MBP treatment (2 g/kg) was initiated immediately after PSNL surgery (day 0) and mechanical allodynia was assessed using the von Frey test (Figure 1). In the PSNL-water group, a significant decrease in withdrawal thresholds was observed in the ipsilateral hind paw compared to the Sham group from days 1 to 21 post-surgery (day 1, *p* < 0.0001 vs. Sham, day 3, *p* < 0.0001 vs. Sham, day 7, *p* = 0.0049 vs. Sham, day 10, *p* = 0.0014 vs. Sham, day 14, *p* = 0.0001 vs. Sham, day 17, *p* < 0.0001 vs. Sham, day 21, *p* = 0.0001 vs. Sham), whereas no such change was detected in the contralateral hind paw (day 1, *p* = 0.6596 vs. Sham, day 3, *p* = 0.9354 vs. Sham, day 7, *p* = 0.1206 vs. Sham, day 10, *p* = 0.0833 vs. Sham, day 14, *p* = 0.2976 vs. Sham, day 17, *p* = 0.2271 vs. Sham, day 21, *p* = 0.105 vs. Sham) (Figure 3). MBP treatment partially but significantly alleviated the PSNL-induced reduction in ipsilateral hind paw withdrawal thresholds on day 7 (*p* = 0.0009 vs. PSNL-Water), day 10 (*p* < 0.0001 vs. PSNL-Water), day 14 (*p* = 0.0009 vs. PSNL-Water), day 17 (*p* = 0.0002 vs. PSNL-Water), and day 21 (*p* < 0.0001 vs. PSNL-Water) (Figure 3). In contrast, no significant differences were observed among the Sham, PSNL-water, and PSNL-MBP groups in the contralateral hind paw throughout the study period (day 1, *p* = 0.9998 vs. PSNL-water, day 3, *p* = 0.7515 vs. PSNL-water, day 7, *p* = 0.1802 vs. PSNL-water, day 10, *p* = 0.1004 vs. PSNL-water, day 14, *p* = 0.2177 vs. PSNL-water, day 17, *p* = 0.1709 vs. PSNL-water, day 21, *p* = 0.1392 vs. PSNL-water) (Figure 3). These findings suggest that MBP has analgesic potential against PSNL-induced neuropathic pain in mice.

### 3.3. MBP Ameliorated the PSNL-Induced Thermal Hyperalgesia in Mice

**Figure 4:**
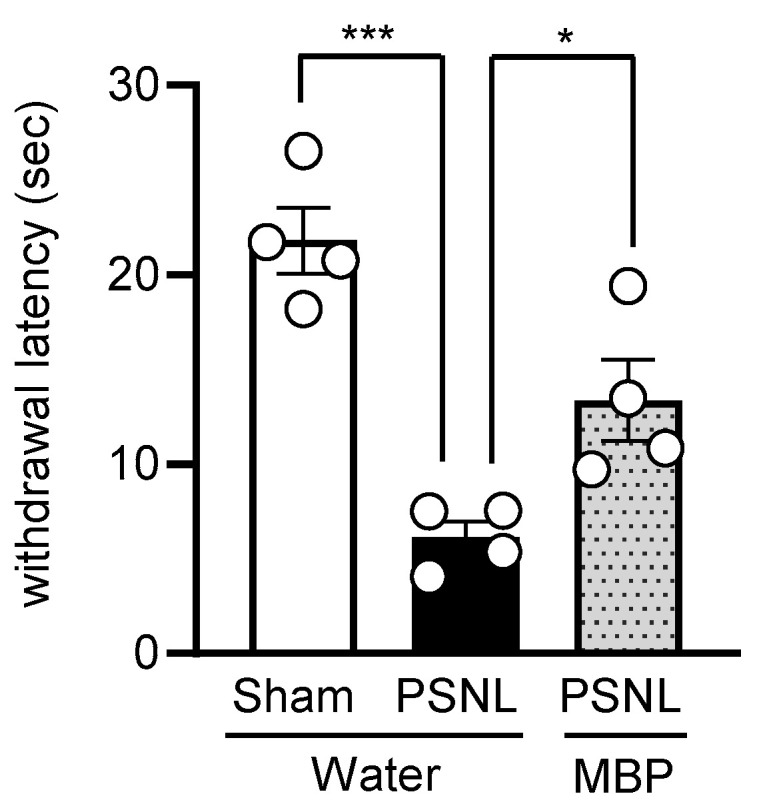
Effect of MBP on thermal hyperalgesia in PSNL mice assessed using the hot plate test. Thermal hyperalgesia was evaluated in the Sham-Water, PSNL-MBP, and PSNL-Water groups on day 7 after surgery. White circles represent individual values. Data are presented as mean ± SE, *n* = 4. Statistical analysis was conducted using one-way ANOVA followed by Tukey’s test. * *p* < 0.05, *** *p* < 0.001.

We evaluated the analgesic effects of MBP on thermal hyperalgesia in PSNL mice using the hot plate test. The test was conducted on day 7 post-surgery (Figure 1). In the PSNL-water group, the withdrawal latency to the thermal stimulus was significantly reduced compared with that in the Sham group (*p* = 0.0003 vs. Sham), indicating the development of thermal hyperalgesia. Oral administration of MBP partially, but significantly, attenuated the PSNL-induced reduction in withdrawal latency (*p* = 0.0332 vs. PSNL-water) (Figure 4). These results indicate that MBP also mitigates PSNL-induced thermal hyperalgesia in mice.

### 3.4. MBP Reduced Microglia Expression in the Dorsal Horn of the Spinal Cord at the Ipsilateral Site of Nerve Injury in PSNL Mice

**Figure 5:**
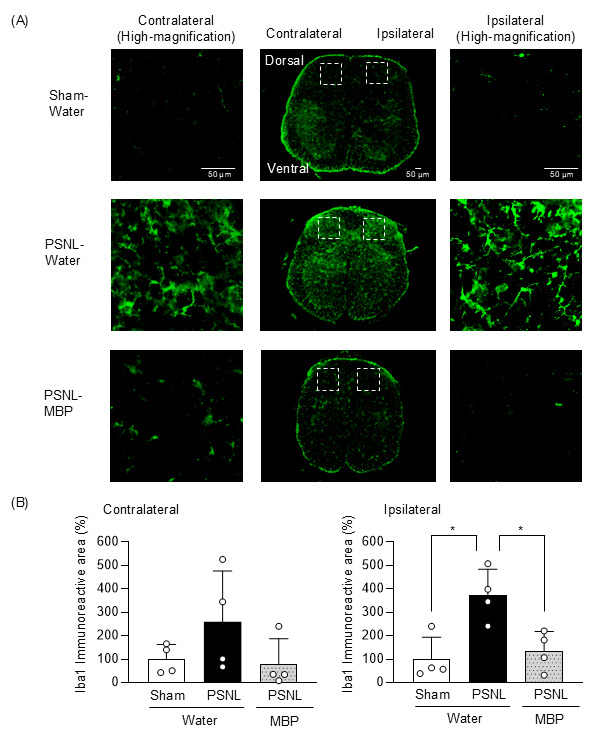
Effect of MBP on increase in the microglial population in the dorsal horn of the spinal cord of nerve injury in PSNL mice. Three days after PSNL surgery, spinal cord segments (L5) were analyzed using immunohistochemistry. (**A**) Representative confocal images show immunofluorescence staining for Iba-1 in the entire spinal cord (L5). The area within the white dashed box in the central panel was captured at high magnification to obtain detailed photographs (right panel: contralateral, left panel: ipsilateral). Scale bars: 50 μm (central panels) and 50 μm (right and left panels). (**B**) The graphs present a semiquantitative analysis of the changes in Iba-1 immunoreactivity in the contralateral (left panel) and ipsilateral (right panel) dorsal spinal cord (L5), expressed relative to the Sham group. Data are shown as mean ± SD, with *n* = 4. Statistical analysis was conducted using non-parametric analysis followed by the two-stage step-up method of Benjamini, Krieger, and Yekutieli. * *p* < 0.05 and q < 0.1.

We conducted an immunohistochemical analysis to assess microglial changes in the SDH of mice on day 3 post-surgery. In the PSNL-water group, Iba-1 immunoreactivity in the ipsilateral dorsal horn was significantly increased compared to the Sham group (*p* = 0.0142, q = 0.0127 vs. Sham). However, MBP treatment significantly attenuated this PSNL-induced increase in Iba-1-positive cells on the ipsilateral side (*p* = 0.0241, q = 0.0127 vs. PSNL-Water group). In the contralateral SDH, a trend toward increased Iba-1 expression was observed in PSNL mice (*p* = 0.3268, q = 0.3431 vs. Sham), which was mitigated by MBP to levels comparable to those in the Sham group (PSNL-MBP, *p* = 0.0499, q = 0.1571 vs PSNL-Water, *p* = 0.3268, q = 0.3431 vs. Sham) (Figure 5). These findings suggested that MBP reduced aberrant microglial activation in the dorsal horn of the spinal cord at the ipsilateral site of nerve injury in PSNL mice.

## 4. Discussion

Neuropathic pain remains a challenging clinical condition due to its complex pathophysiology and limited responsiveness to conventional pharmacological treatments. In our previous study, oral administration of MBP after the onset of ALS-like symptoms prolonged survival by suppressing astrocyte and microglial activation in the spinal cord of G93A mice [14]. In this study, we demonstrated for the first time that MBP significantly and sustainably ameliorated mechanical allodynia in PSNL mice. We also found that MBP suppressed microglial expression in the lumbar spinal cord of PSNL mice. Our results indicate that MBP may be a potential therapeutic agent for the treatment of neuropathic pain by restoring the balance of microglia in the spinal cord.

MBP and BP have been traditionally used as ethnomedicines and functional foods in various regions. Prior research has indicated that the oral administration of BP extract at 400 mg/kg body weight, divided into three doses per day, for three months did not cause any noticeable toxicity in humans [20]. An acute toxicity study in mice determined the median lethal dose (LD50) of the BP water extract to be 12.3 g/kg body weight [21]. In rats, daily oral intake of the BP water extract at a maximum dose of 1 g/kg body weight for 4 weeks (28 days) showed no significant adverse effects, with no fatalities or abnormalities observed in the survival rate, body weight, or macroscopic organ analysis [22]. In animal studies, a BP dose of 27 g/kg body weight (at a dose of 2.5% food) for 24 weeks (168 days) demonstrated anti-obesity and anti-diabetic properties in leptin-deficient (ob/ob) mice, without any evident toxic effects [23]. Mice receiving a diet containing 10% BP for 24 weeks (168 days) displayed slight toxicological effects, such as decreased food intake and lower body weight gain [24]. However, BP at concentrations of ≤5% for 24 weeks (168 days) was well-tolerated, as indicated by the evaluation of survival rate, body and organ weight, dietary and water intake, serum biochemical markers, urinalysis, hematological parameters, organ histology, and genotoxicity assessments [24]. Moreover, we found that neither wild-type nor G93A mice exhibited toxicity in the spinal cord tissue, weight loss, or abnormal behavior after receiving MBP at 2 g/kg body weight daily for approximately 40 days [14]. In the present study, MBP administration at the same dose for 21 days did not influence weight gain in PSNL mice, and no significant differences were observed compared to the sham-operated mice. These results indicate that MBP was well-tolerated in multiple mouse models, supporting its potential for further investigation in human studies.

Several lines of evidence indicate that microglial activation is a key pathological factor in neuropathic pain [25,26]. Therefore, recent studies have emphasized the potential of spinal microglial activation inhibitors as therapeutic agents for treating neuropathic pain. Indeed, treatment with pexidartinib (PLX3397), an inhibitor of the colony-stimulating factor 1 receptor, significantly suppressed mechanical allodynia and reduced the activation of Iba-1 positive microglia in the dorsal horn of the lumbar spinal cord following PSNL [27]. More recently, we demonstrated that the post-onset intranasal administration of N-acetyl-L-cysteine (NAC) combined with cell-penetrating peptide-modified polymer micelles ameliorated mechanical allodynia by suppressing microglial proliferation in PSNL mice [16]. The ethanolic extract of *Bauhinia brachycarpa* Benth [26], a traditional Chinese herbal medicine, was reported to improve PSNL-induced mechanical allodynia by regulating microglial activation in the lumbar spinal cord of mice. In this study, immunohistochemical analysis was performed on day 3 post-surgery to capture the early cellular events following PSNL. This time point was chosen based on previous studies indicating that microglial expression in the spinal cord peaks within 3 days after nerve injury [28]. This rationale is further supported by recent findings showing that microglia-mediated degradation of perineuronal nets is also initiated on day 3 post-injury and persists for at least two weeks, indicating that early microglial responses play a critical role in shaping persistent pain states [29]. We found that MBP significantly suppressed the PSNL-induced increase in Iba-1-positive cells on the ipsilateral side (Figure 5), which indicated that MBP inhibited microglial activation in the spinal cord of PNSL mice. Furthermore, in a human ALS-linked mutant superoxide dismutase-1 (SOD1^G93A^) transgenic mouse model of ALS (G93A mice), suppression of microglial/macrophage proliferation apparently contributes to the neuroprotective effects of MBP on motor neurons in the lumbar spinal cord [14]. We observed a marked reduction in mechanical allodynia and thermal hyperalgesia following MBP administration (Figure 3 and Figure 4). These findings suggest that the therapeutic effects of MBP on neuropathic pain may be mediated, at least in part, by inhibiting Iba1-positive microglial expression. However, to fully characterize the nature of this microglial modulation, the characteristics of the markers employed should be considered. Despite being a widely used marker for microglia, Iba1 does not allow for a clear distinction between changes in proliferation and the activation state. In addition, while previous studies have sometimes used the M1/M2 framework to describe microglial phenotypes, this binary classification is now considered an oversimplification, as microglial activation is increasingly understood as a dynamic and context-dependent spectrum of functional states [30]. Additional markers such as CD68, TMEM119, or MHC-II were excluded from this study, thereby limiting the interpretation of microglial dynamics. The aim of future studies should be to improve the characterization of the microglial response to MBP treatment by incorporating multiple markers, and specifically to clarify how MBP influences distinct microglial states within this activation spectrum in the context of neuropathic pain.

One limitation of this study was that we could not identify the specific bioactive compounds responsible for the anti-inflammatory and antinociceptive effects of MBP. Previous studies have shown that MBP contains caffeic acid, chlorogenic acids, such as neochlorogenic acid and chlorogenic acid, and multiple flavonoids, including rutin, quercetin, hyperin, isoquercitrin, centaurein, and jacein [31,32]. Chlorogenic acid and caffeic acid have been reported to alleviate neuropathic pain in CCI models [33,34]. In addition, chlorogenic acid has been shown to inhibit lipopolysaccharide-induced activation of primary mouse microglia [35]. It is plausible that these bioactive components interact synergistically to modulate microglia-driven inflammation in the spinal cords of PSNL mice. Moreover, the precise mechanisms underlying microglial inhibition in MBP-treated PSNL mice remain unclear. Previous studies have shown that Toll-like receptor 4 (TLR4) activation in microglia contributes to the development of neuropathic pain through the release of pro-inflammatory cytokines [36]. The pharmacological inhibition of TLR4, such as with epigallocatechin gallate or (−)-naloxone, has been reported to attenuate pain behaviors in rodent models [37,38]. Therefore, MBP may suppress the production of pro-inflammatory cytokines by inhibiting microglial expression. In this study, MBP was evaluated at only a single dose (2 g/kg/day) because of its limited solubility (0.1 g/mL) and administration volume (0.2 mL/10 g body weight). Although no significant loss in body weight or overt signs of systemic toxicity were observed through Day 21 (Figure 1 and Figure 2), the lack of a dose-response analysis limits the interpretation of the effective dose range. Further research is required to elucidate the specific active compounds and mechanisms underlying the effects of MBP, as well as to characterize the pharmacological profile across a broader dose range. Nevertheless, leveraging ethnomedicine and functional foods to develop anti-inflammatory therapeutics targeting microglia-mediated inflammation may represent a promising strategy for treating neuropathic pain.

## 5. Conclusions

**Figure 6:**
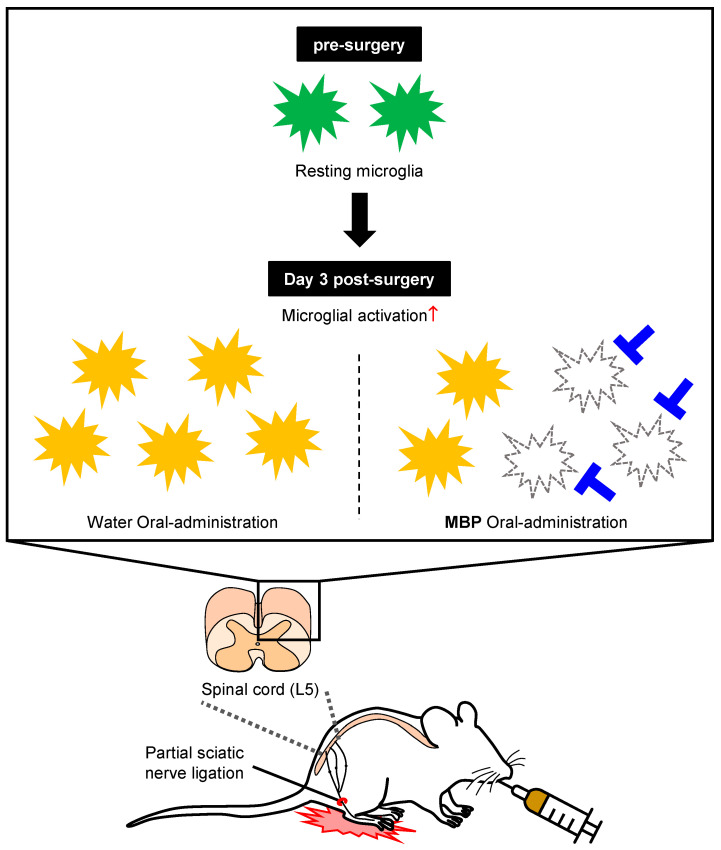
Proposed mechanism for the antinociceptive effects of MBP in the spinal cord of a neuropathic pain model mouse.

In conclusion, this study provides the first evidence that the oral administration of MBP alleviates mechanical allodynia in a PSNL-induced neuropathic pain model by inhibiting microglial activation in the SDH (Figure 6). However, further studies are required to elucidate the molecular mechanisms underlying these effects. These findings suggest that MBP is a potential therapeutic option for the treatment of neuropathic pain associated with microglial activation. Given its traditional use as a functional food and its demonstrated efficacy in modulating microglia-mediated neuroinflammation, MBP may offer a novel and practical therapeutic avenue for neuropathic pain management with a favorable safety profile.

## Data Availability

The original contributions of this study are included in this article. Further inquiries can be directed to the corresponding author.
